# Reconsideration of the Semaphorin-3A Binding Motif Found in Chondroitin Sulfate Using *Galnac4s-6st*-Knockout Mice

**DOI:** 10.3390/biom10111499

**Published:** 2020-10-30

**Authors:** Satomi Nadanaka, Shinji Miyata, Bai Yaqiang, Jun-ichi Tamura, Osami Habuchi, Hiroshi Kitagawa

**Affiliations:** 1 Laboratory of Biochemistry, Kobe Pharmaceutical University, Kobe 658-8558, Japan; snadanak@kobepharma-u.ac.jp (S.N.); baiya966@st.kobepharma-u.ac.jp (B.Y.); 2 Faculty of Agriculture, Tokyo University of Agriculture and Technology, Tokyo 183-8509, Japan; smiyata@go.tuat.ac.jp; 3 Department of Agricultural, Life and Environmental Sciences, Faculty of Agriculture, Tottori University, Tottori 680-8553, Japan; jtamura@tottori-u.ac.jp; 4 Multidisciplinary Pain Center, Aichi Medical University, Yazako, Nagakute, Aichi 480-1195, Japan; ohabuchi@aichi-med-u.ac.jp

**Keywords:** chondroitin sulfate, proteoglycans, perineuronal net, extracellular matrix

## Abstract

The chondroitin sulfate (CS)-rich dense extracellular matrix surrounding neuron cell bodies and proximal dendrites in a mesh-like structure is called a perineuronal net (PNN). CS chains in PNNs control neuronal plasticity by binding to PNN effectors, semaphorin-3A (Sema3A) and orthodenticle homeobox 2. Sema3A recognizes CS-containing type-E disaccharide units (sulfated at O-4 and O-6 of *N*-acetylgalactosamine). Type-E disaccharide units are synthesized by *N*-acetylgalactosamine 4-sulfate 6-*O*-sulfotransferase (GalNAc4S-6ST). In this study, we demonstrated that Sema3A accumulates in the PNNs surrounding parvalbumin cells, even in mice deficient in GalNAc4S-6ST. In addition, there were no differences in the number and structure of PNNs visualized by Cat316 antibody and *Wisteria floribunda* lectin, which recognize CS chains, between wild type and GalNAc4S-6ST knockout mice. Therefore, we re-examined the Sema3A binding motif found in CS chains using chemically synthesized CS tetrasaccharides. As a result, we found that non-sulfated GalNAc residues at the non-reducing termini of CS chains are required for the binding of Sema3A.

## 1. Introduction

Individual brain regions are highly plastic in response to environmental stimuli during early developmental processes, termed critical periods [1]. During critical periods, neural circuits in the brain can be reorganized based on new experiences. However, the nature of the critical period is currently poorly understood. At the cellular level, GABA neurons expressing parvalbumin (PV) are likely candidates for controlling timing-critical period plasticity [2], and the maturation of PV neurons has been linked to the onset of the critical period [3,4,5]. At the network level, experience-dependent changes in brain inhibitory circuits formed by PV cells are thought to play a key role during the critical period of brain development.

As PV cells mature, they are enwrapped by a specialized extracellular matrix called a perineuronal net (PNN) that interdigitates with synaptic contacts. During postnatal development, PNNs appear on PV neurons and reach mature levels by the end of the critical period [6,7]. Although the function of PNNs has not yet been clarified, it has been shown to exert neuroplasticity control and have neuroprotective effects. Accumulation of PNNs in PV cells is involved in both the onset and closure of the critical period. In animal models, removal or digestion of PNNs increases plasticity, leading to enhanced memory interference from competing information during the encoding process [6,8,9]. These findings suggest that the deposition of PNN tightly enwrapped PV neurons accelerates the maturation of PV neurons and is linked to the end of critical period plasticity.

Functional abnormalities in PV neurons may be a cause of psychiatric diseases, including depression, autism, and schizophrenia [10]. PV neurons control the timing of pyramidal cell output by forming highly interconnected networks of cortical neurons and regulating the balance between excitatory and inhibitory neurotransmission (E/I balance). These functions of PV neurons are involved in active cognition, learning, and memory. Therefore, defects in the functions of PV cells and circuits that they form have been implicated in mental disorders [11]. On the other hand, PNNs may be involved in some psychological disorders, such as schizophrenia, as the number of PNNs has been found to be reduced in patients with this disorder [12,13]. These findings suggest that the functions of PV neurons and PNN formation are associated with psychiatric diseases.

Chondroitin sulfate proteoglycans (CS-PGs) accumulate around PV neurons, forming PNNs. Digestion of CSs with chondroitinase ABC results in PNN disruption, suggesting the involvement of CS chains in PNN formation. In addition, PNNs can modify PV cell function by facilitating the sequestration of secreted proteins at the neuronal surface via interactions with CS chains [14]. These interactions are regulated by the fine structure of the CS chains [15,16,17]. CS chains, linear polysaccharides consisting of repeating disaccharide units [-4GlcAβ1-3GalNAcβ1]_n_], are linked to serine residues in core proteins [13]. The chondroitin backbone, consisting of repeating disaccharide units, may be modified with sulfate at positions 4 and 6 of GalNAc residues and position 2 of GlcA residues. In the first step, a non-sulfated O unit (GlcA-GalNAc) is modified by either chondroitin 4-*O*-sulfotransferase (C4ST) or C6ST, resulting in the formation of the A [GlcA-GalNAc(4-*O*-sulfate)] or C [GlcA-GalNAc(6-*O*-sulfate)] unit, respectively. Subsequently, a small portion of A units are converted to E units [GlcA-GalNAc(4,6-*O*-disulfate)] by GalNAc 4-sulfate 6-*O*-sulfotransferase (GalNAc4S-6ST), which catalyzes the 6*-O*-sulfation of GalNAc(4-*O*-sulfate). On the other hand, some C units are converted to D units [GlcA(2-*O*-sulfate)-GalNAc(6-*O*-sulfate)] by uronyl 2-*O*-sulfotransferase, which catalyzes the 2*-O*-sulfation of GlcA. The arrangement of these sulfated units along with the CS chains creates sulfation codes that may convey the functional information carried by CS-PGs. Sulfation codes of CS chains regulate neural plasticity through specific interactions of CS chains with its binding partner proteins, such as orthodenticle homeobox 2 (OTX2) and semaphorin 3A (Sema3A) [18,19].

The interaction between PNN and its binding partner proteins is linked to cognitive and cortical plasticity, and its defect is associated with schizophrenia. Therefore, it is important to determine the binding sites of CS chains. Beurdeley et al. reported that the sulfation code, including C or E units, mediates OTX2 binding to PNNs [19,20]. The Sema3A binding site of CS chains is controversial. Dick et al. showed that Sema3A binds to PNNs via E unit-containing CS chains [18]. In contrast, Yang et al. demonstrated that A unit-containing CS chains function as a binding site for Sema3A [21]. Here, we re-examined the Sema3A binding site of CS chains and determined whether E units are required for the binding of Sema3A to PNNs.

## 2. Materials and Methods

### 2.1. Enzyme-Linked Immunosorbent Assay

After Nunc Immobilizer^TM^ Streptavidin was washed with phosphate buffered saline (PBS) containing 0.1% Tween 20 (PBST), biotinylated chemically synthesized tetrasaccharides [22,23] or biotinylated CS polymers were added to each well and incubated for 1 h at 25 °C. CS-A from whale cartilage, CS-C and CS-D from shark cartilage, CS-E from squid cartilage, and heparin from porcine intestine (Seikagaku Corporation, Tokyo, Japan) were biotinylated as previously reported [24]. O-O tetrasaccharides (0.5 μg/well) immobilized in each well were digested with β-hexosaminidase (β-HexNAcase) (2.5 units/well) (Cat. No. P0721S, New England BioLabs Inc., Ipswich, MA, USA) for 24 h at 37 °C. Alternatively, O-O tetrasaccharides (5 μg) were digested with β-HexNAcase (25 units/well) for 24 h at 37 °C. After washing with PBST, each well was blocked with Blocking One (Nacalai Tesque, Inc., Kyoto, Japan) for 1 h at room temperature. The contents of each well were reacted with Cat316 (dilution 1/5000; MAB1582, Sigma-Aldrich, St. Louis, MO, USA) or horseradish peroxidase-conjugated *Wisteria floribunda* lectin (WFA; dilution 1/2500; Cat. No. H-3101-1, EY laboratories, Inc., San Mateo, CA, USA) overnight at 4 °C. For the detection of Cat316-binding, horseradish peroxidase-conjugated anti-mouse IgM antibody (dilution 1/10,000) was added to each well after washing with PBST and incubated for 2 h. Next, 2,2′-azino-bis(3-ethylbenzothiazoline-6-sulphonic acid) was added to each well as a peroxidase substrate. For the detection of WFA binding, 2,2′-azino-bis(3-ethylbenzothiazoline-6-sulphonic acid) was added to each well as a peroxidase substrate. The blue-green-colored materials produced by peroxidase were measured at 405 nm.

To inhibit the binding of Cat316 and WFA lectin to O-O tetrasaccharides using Sema3A as an inhibitor, recombinant human Sema3A/Fc chimera proteins (Cat. No. 1250-S3, R&D Systems, Inc., Minneapolis, MN, USA) were used. After O-O tetrasaccharides were immobilized in each well, Sema3A proteins (50 μl/well) were added to each well at the indicated concentration and incubated at 4 °C overnight. To detect the binding of recombinant Sema3A proteins to glycosaminoglycan (GAG) polymers and CS tetrasaccharides, anti-Sema3A antibody (dilution 1/100; sc-74554, SantaCruz, Dallas, TX, USA) was used.

### 2.2. Animals

*Galnac4s-6st* knockout mice have been described previously [25]. Mice were kept under pathogen-free conditions in an environmentally-controlled, clean room at the Institute of Laboratory Animals, Kobe Pharmaceutical University. Animals were maintained on standard rodent food and a 12 h light/dark cycle. All animal procedures were approved by the Kobe Pharmaceutical University Committee on Animal Research and Ethics (2019-037, 9 May 2019; 2020-042, 16 April 2020).

### 2.3. Real-Time PCR Analysis

Total RNA was isolated from the whole brain using a Maxwell^®^ 16 LEV simplyRNA Purification Kit (Promega, Madison, WI, USA). Aliquots (0.5 μg) of total RNA digested with DNase were treated with M-MLV reverse transcriptase (Invitrogen, Carlsbad, CA, USA) using random primers (nonadeoxyribonucleotide mixture; pd(N)_9_; Takara Bio Inc., Shiga, Japan). Quantitative real-time PCR was conducted using Thunderbird^®^ Probe qPCR Mix (TOYOBO, Osaka, Japan) and a LightCycler^®^ 96 System (Roche Applied Science, Upper Bavaria, Germany). The housekeeping gene *glyceraldehyde-3-phosphate dehydrogenase* was used as an internal control for quantification. The primers used for real-time PCR were purchased from Applied Biosystems TaqMan^TM^ Assays (Thermo Fisher Scientific, Inc., Waltham, MA, USA), and a list of these primers is provided in Table 1.

### 2.4. Immunohistochemistry

Under sodium pentobarbital anesthesia (Somnopentyl, Kyoritsu Seiyaku, Tokyo, Japan), 10-week-old male mice were fixed using the perfusion-fixation method [23], and their brains were removed. Brains were post-fixed overnight with 4% paraformaldehyde in PBS. Coronal sections (30 μm thick) were cut with a vibratome (LEICA Microsystems, Wetzlar, Germany). Sections were permeabilized with 0.2% Triton X-100 in PBS for 15 min and incubated overnight at 4 °C with the primary antibodies and WFA as follows: anti-PV antibody (dilution: 1/2000; Cat. No. 235, SWANT^®^ Swiss antibodies, Marly, Switzerland), Cat316 (dilution: 1/2000; Cat. No. MAB1582, Sigma-Aldrich, St. Louis, MO, USA), and biotinylated WFA (dilution: 1/400; Cat. No. B-1355; Vector Laboratories, Inc., Burlingame, CA, USA).

Images were acquired using a Zeiss LSM 700 confocal laser scanning system (Carl Zeiss Inc., Oberkochen, Germany) equipped with an inverted Axio observer Z1 microscope, Objective Plan-Apochromat 10x/0.45, Objective Plan-Apochromat 20x/0.8, and Objective Plan-Apochromat 63x/1.3 Oil. Subsequent image analysis was performed using the LSM software ZEN 2009 (Carl Zeiss Inc., Oberkochen, Germany). Wide-field-of-view microscopic cortical images were acquired using zoom microscopy Axio Zoom V16 (Carl Zeiss Inc. Oberkochen, Germany).

### 2.5. Immunoblotting

Extraction of PGs from the brain was carried out as described previously [26]. The brain lysates (600 μg of proteins) were digested with 5 milliunits of chondroitinase ABC (EC4.2.2.4; Seikagaku Corporation, Tokyo, Japan) and 5 units of β-HexNAcase for 2 h at 37 °C. Undigested and chondroitinase ABC-digested lysates (40 μg of proteins) were separated by Bullet PAGE One Precast Gel 5–20% (Nacalai Tesque Inc., Kyoto, Japan). Immunoblotting was performed using the following primary antibodies, as described previously [16]: anti-aggrecan antibody (dilution: 1/2000; AB1031, Merck Millipore, Burlington, MA, USA), anti-brevican antibody (Cat. No. RUO-610894, BD Biosciences, San Jose, CA, USA), anti-phosphacan antibody (dilution: 1/200; clone 3F8, DHSB, Iowa, IA, USA), Cat316 (dilution:1/10,000), 1B5 (dilution: 1/10,000; Cat. No. 270431, Sekagaku Business Corp., Tokyo, Japan), and 2B6 (dilution: 1/10,000; Cat. No. 270432, Sekagaku Business Corp., Tokyo, Japan).

### 2.6. Statistical Analysis

Data are expressed as the mean ± one standard deviation of the mean. Statistical significance was determined by Student’s *t*-test.

## 3. Results

### 3.1. Determination of the Epitope of the Cat316 Antibody

**Figure 1:**
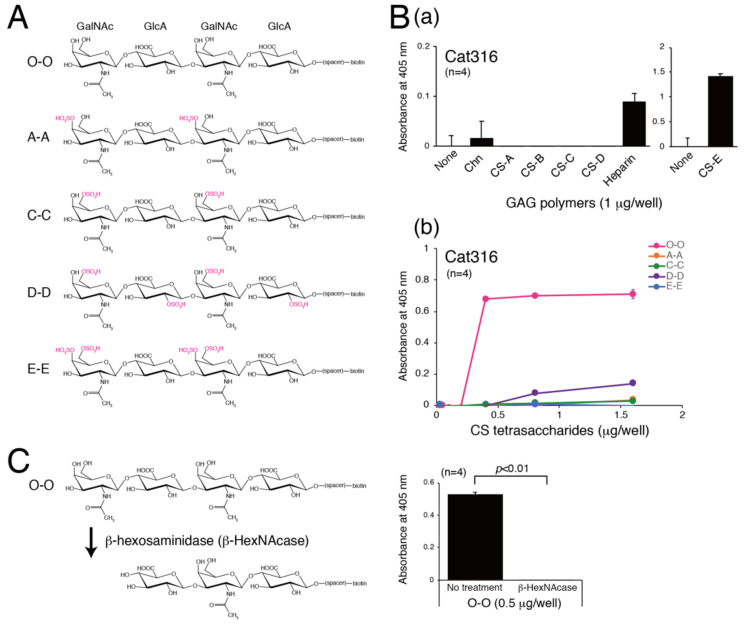
Cat316 specifically recognizes non-sulfated terminal GalNAc residues. (**A**) Structures of chemically synthesized biotinylated tetrasaccharides with various sulfations. (**B**) Biotinylated chondroitin (Chn) and GAG polymers (CS-A, CS-B, CS-C, CS-D, and heparin) (**a**) and chemically synthesized biotinylated tetrasaccharides (O-O, A-A, C-C, D-D, and E-E) (**b**) were immobilized onto a streptavidin-coated plate, and Cat316 was added to each well. Cat316 binding to immobilized GAGs or tetrasaccharides was measured by ELISA. (**C**), Cat316 binding to O-O tetrasaccharides digested with or without β-hexosaminidase (β-HexNAcase) was examined. Statistical significance was determined using Student’s *t*-test.

In a study, Cat316 was reported to bind to the CS chains on aggrecans [27]. In addition, Yang et al. found that Cat316 inhibits the binding of Sema3A to aggrecans [21]. These findings indicate that Cat316 recognizes CS structures involved in the interaction with Sema3A. Here, we examined the epitope of Cat316 using chemically synthesized tetrasaccharides, as shown in Figure 1A. Five types of tetrasaccharides (O-O, A-A, C-C, D-D, and E-E) were biotinylated at the reducing terminal and modified with various sulfation patterns [22,23]. First, the binding of Cat316 to biotinylated CS polymers was examined, and Cat316 was found to bind to heparin and CS-E (Figure 1B(a)). We next examined the binding of Cat316 to five types of tetrasaccharides. Unexpectedly, Cat316 bound to non-sulfated tetrasaccharides, O-O, with high affinity (Figure 1B(b)). Furthermore, removal of the GalNAc residue located at the non-reducing terminal by digestion with β-hexosaminidase (β-HexNAcase) dramatically reduced the interaction with Cat316 (Figure 1C). These results suggest that Cat316 specifically recognizes non-reducing terminal non-sulfated GalNAc residues. 

### 3.2. Determination of Structural Requirements for Recognition by WFA

**Figure 2:**
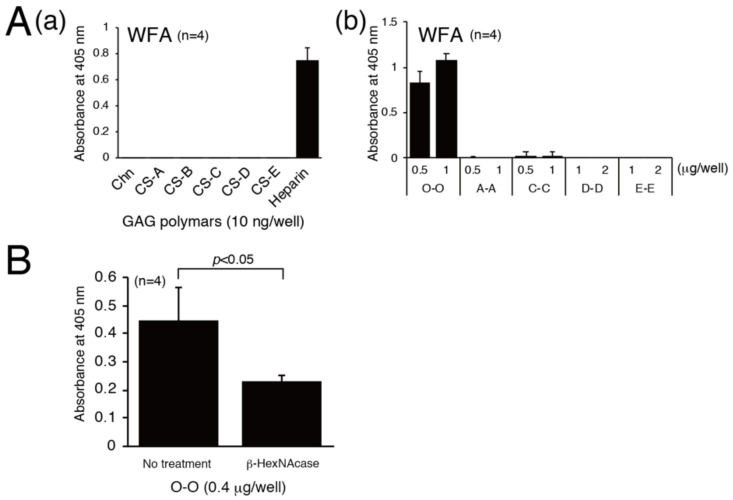
WFA specifically recognize non-sulfated tetrasaccharides (**A**), Biotinylated Chn and GAG polymers (CS-A, CS-B, CS-C, CS-D, and heparin) (**a**) and chemically synthesized biotinylated tetrasaccharides (O-O, A-A, C-C, D-D, and E-E) (**b**) were immobilized onto a streptavidin-coated plate, then horseradish-conjugated WFA was added to each well. WFA binding to immobilized GAGs or tetrasaccharides was measured by ELISA. (**B**) WFA binding to O-O tetrasaccharides digested with or without β-HexNAcase was examined.

WFA reacts to glycans containing terminal GalNAc residues. Aggrecan-deficient neurons lack staining for WFA, indicating that WFA recognizes CS chains carried by aggrecan, a major component of PNNs. We examined the specific structure involved in the binding of WFA because it can be used as a probe to detect the CS structure involved in the interaction with its binding partner proteins, such as Sema3A and the OTX2 homeoprotein. We assessed the binding of WFA to GAG polymers and found that WFA bound only to heparin (Figure 2A(a)). Of the five types of tetrasaccharides, WFA specifically interacted with non-sulfated tetrasaccharides, O-O (Figure 2A(b)). As shown in Figure 2B, digestion of O-O tetrasaccharides with β-HexNAcase partially diminished their binding to WFA. Therefore, WFA lectin preferentially recognizes non-reducing terminal GalNAc residues, although WFA lectin can bind to both non-reducing terminal and internal GalNAc residues.

### 3.3. Examination of the Sema3A-Binding Motif

**Figure 3:**
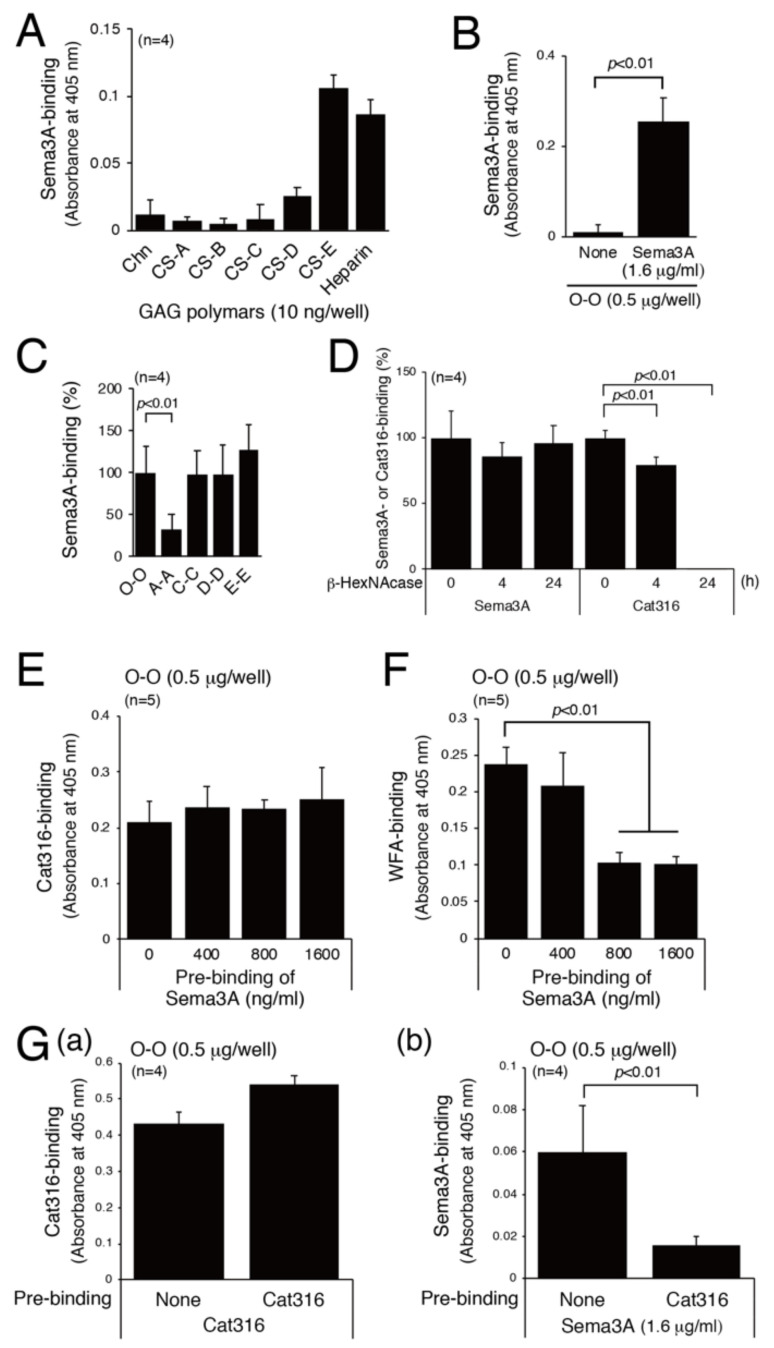
The Sema3A binding motif is partially recognized by Cat316 and WFA. (**A**) Binding of Sema3A to biotinylated Chn and GAG polymers (CS-A, CS-B, CS-C, CS-D, and heparin) was examined by ELISA. (**B**) Binding of Sema3A to biotinylated tetrasaccharides, O-O, was examined by ELISA. (**C**) Binding of Sema3A to A-A, C-C, D-D, and E-E tetrasaccharides is shown as a percentage of the binding of Sema3A to O-O tetrasaccharides. (**D**) Binding of Sema3A and Cat316 to O-O tetrasaccharides was examined after O-O tetrasaccharides were digested with β-HexNAcase for 0, 4, and 24 h. (**E**) Binding of Cat316 to O-O tetrasaccharides was examined after O-O tetrasaccharides were incubated with Sema3A (0, 400, 800, and 1600 ng/mL). (**F**) Binding of WFA to O-O tetrasaccharides was inhibited using Sema3A (0, 400, 800, and 1600 ng/mL) as a competitor. (**G**) The effect of Cat316 pretreatment on the binding of Cat316 (**a**) and Sema3A (**b**).

As reported previously [18], Sema3A binds to CS-E and heparin (Figure 3A). In addition, Sema3A interacts with O-O, C-C, D-D, and E-E tetrasaccharides (Figure 3B,C). We assessed whether the interaction between Sema3A and CS-E is physiologically significant. The structural requirements for Sema3A-binding were also examined, taking advantage of the binding specificity of Cat316 and WFA. As shown in Figure 3D, Cat316 did not bind to O-O tetrasaccharides digested with β-HexNAcase, whereas binding of Sema3A to O-O tetrasaccharides was not affected by this digestion. We next examined whether Sema3A and Cat316 share the same binding site. Pre-binding of Sema3A to O-O tetrasaccharides hardly affected Cat316-binding to O-O tetrasaccharides (Figure 3E). Compared with Cat316, WFA binding to O-O tetrasaccharides was inhibited by pre-binding of Sema3A to O-O tetrasaccharides in a concentration-dependent manner (Figure 3F).

In contrast with Figure 3E, pre-binding of Cat316 with O-O tetrasaccharides effectively inhibited the binding of Sema3A to O-O saccharides (Figure 3G). The discrepancy between the results shown in Figure 3E,G(b) may be explained as follows: because the non-reducing terminal GalNAc residue might not be masked by Sema3A, Cat316 can bind to O-O tetrasaccharides even after Sema3A binds to O-O saccharides Figure 3E. In contrast, Sema3A could not access the binding site in O-O tetrasaccharides because Cat316, an IgM pentamer, is bulky Figure 3G(b). These results suggest that Sema3A binds to CS structures recognized by Cat316 and WFA, but their binding motif does not completely overlap. Sema3A may bind to non-sulfated CS structures located adjacent to the non-reducing terminus (see the “Discussion” section).

### 3.4. Analysis of Physiological Significance of the Binding of Sema3A to CS-E

**Figure 4:**
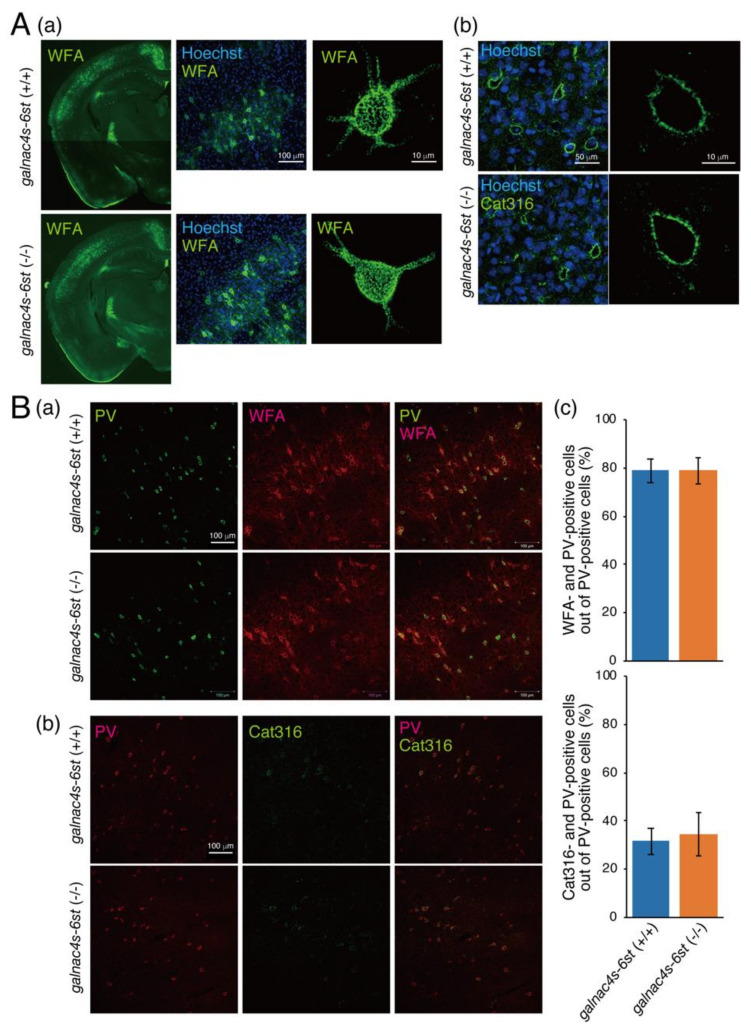
PNNs formed in *galnac4s-6st*(−/−) mice are comparable to those in *galnac4s-6st*(+/+) mice. (**A**) Coronal sections from the brains of 10-week-old *galnac4s-6st*(+/+) and *galnac4s-6st*(−/−) mice were stained with WFA (**a**) and Cat316 mAb (**b**). WFA-positive PNNs were visualized in Z-section using confocal microscopy. (**B**) PV cells and WFA-positive PNNs (**a**) as well as Cat316-positive PNNs (**b**) were immunohistochemically detected. (**c**) The percentage of PV neurons surrounded by WFA- or Cat316-positive PNNs out of PV-positive neurons was quantified.

We examined whether Sema3A requires CS-E structures (E unit) for binding to CS chains in PNNs using mice lacking GalNAc4S-6ST responsible for biosynthesis of a biosynthetic enzyme of the E unit. The distribution of WFA-positive PNNs, fluorescent intensity of WFA staining, and meshwork surrounding the soma were not affected by the loss of the E unit (Figure 4A(a),B(a)). In addition, the fluorescent intensity of Cat316-staining was not affected by the loss of E units (Figure 4A(b),B(b)). The percentage of WFA- and Cat316-positive PNNs formed around PV neurons was investigated. Most PV neurons were surrounded by WFA-positive PNN in both *galnac4s-6st* (+/+) and *galnac4s-6st* (−/−) mice (Figure 4B(c)). In addition, the percentage of PV neurons surrounded by Cat316-positive PNNs out of PV-positive neurons was not affected by the loss of E units (Figure 4B(c)). 

**Figure 5:**
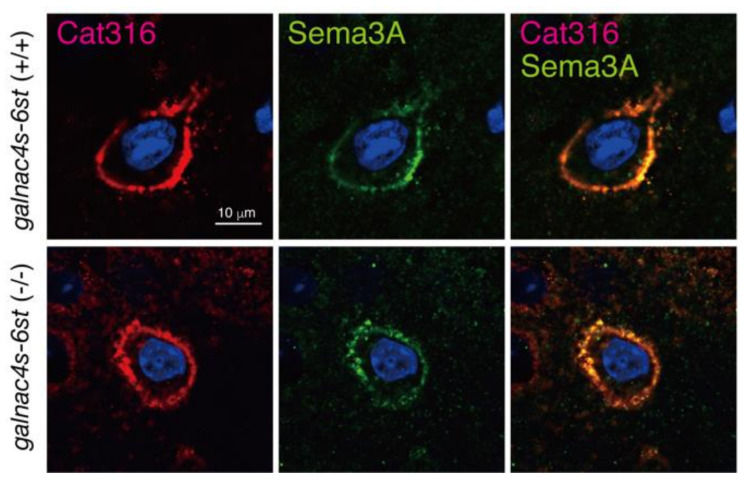
Sema3A is co-localized in Cat316-positive CSs even in the absence of E units Coronal sections from the brains of 10-week-old *galnac4s-6st* (+/+) and *galnac4s-6st* (−/−) mice were stained with Cat316 mAb and anti-Sema3A antibody. Sema3A proteins also accumulated in E units-deficient PNNs.

The expression levels of proteoglycans (PGs), major components of PNN, were compared between *galnac4s-6st* (+/+) and *galnac4s-6st* (−/−) mice (Figure S1). Compared with *galnac4s-6st* (+/+) mice, aggrecan (Acan), phosphacan (Pcan), and brevican (Bcan) were expressed at similar levels in *galnac4s-6st* (−/−) mice (Figure S1A). The expression levels of Cat316-reactive CS chains were similar in *galnac4s-6st* (−/−) mice to those of *galnac4s-6st* (+/+) mice (Figure S1A(b)). Consistent with a previous study [26], Cat316 reactivity was diminished by chondroitinase ABC and β-HexNAcase (Figure S1A(b)). Monoclonal antibodies 1D5 and 2B6 recognize CS structures that are newly generated after digestion with chondroitinase ABC. The expression levels of 1D5- and 2B6-reactive CS chains were not affected by the loss of E units (Figure S1A(b)). In addition, the gene expression levels of *Acan*, *Bcan*, *neurocan* (*Ncan*), *versican* (*Vcan*), *Pcan*, and *syndecan 3* were examined by real-time PCR. We found no differences in the expression levels of these genes between *galnac4s-6st* (−/−) and *galnac4s-6st* (+/+) mice (Figure S1B). Furthermore, we examined whether Cat316-positive and E-unit-negative PNNs could capture Sema3A molecules. As shown in Figure 5, deposition of Sema3A was observed in Cat316-positive PNNs of *galnac4s-6st* (−/−) mice. These results suggest little or no effect of E units on PNN formation and its binding property to PNN effectors. 

## 4. Discussion

Although it has been suggested that CS chains in the PNNs control neuronal plasticity through the binding of PNN effectors such as Sema3A and OTX2, the binding properties of CS chains to these effector proteins remain unclear. Previous studies have demonstrated that Sema3A selectively binds to CS chains containing E units in vitro [18]. Our analysis indicates that CS chains containing PNNs are produced by PV neurons [28]. In addition, it has been found that the *galnac4s-6st* gene is barely expressed in PV neurons through analysis using a single-cell RNA-seq method [29]. These findings prompted us to reconsider the CS structures recognized by Sema3A. Here, we examined the physiological significance of the interaction between Sema3A and E units using *galnac4s-6st* (−/−) mice. As a result, we found that PNNs containing CS chains lacking the E unit could capture Sema3A molecules. Therefore, we attempted to identify the CS structure involved in the interaction with Sema3A using an approach from a different angle. Sema3A was co-localized with Cat316-positive CS chains (Figure 5), suggesting the selective binding of Sema3A to Cat316-positive CS chains. In addition, WFA recognizes CS chains carried by aggrecans, a major component of PNNs, because primary cortical neurons from aggrecan-deficient mice apparently lack staining for WFA [27,30]. Some potential mediators of PNN-controlled plasticity such as OTX2 and Sema3A may be bound to the CS chains of PGs, which are most likely aggrecans [14,20,31]. Therefore, we determined the CS structures recognized by Cat316 and WFA. Here, we show both Cat316 and WFA preferentially bound to non-sulfated CS tetrasaccharides. Furthermore, Cat316 strictly recognized non-sulfated GalNAc residues at the non-reducing terminal, whereas WFA bound non-sulfated GalNAc residues located adjacent to the non-reducing terminus.

**Figure 6:**
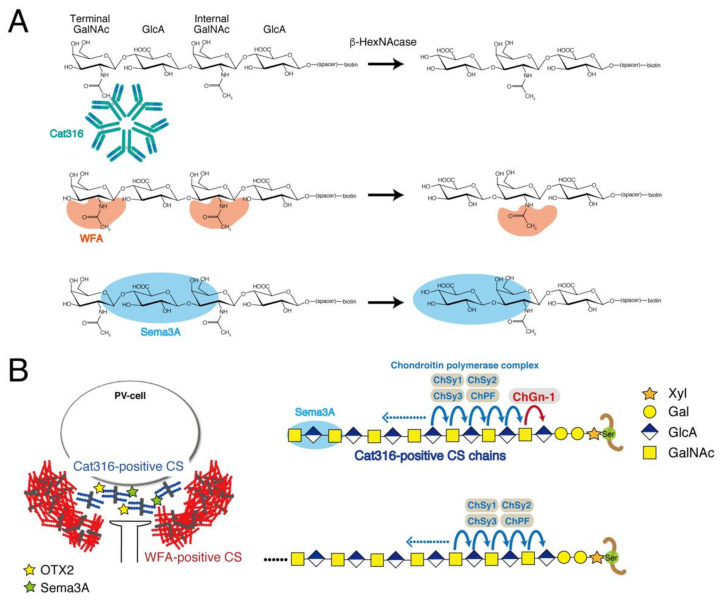
Structural requirements for the binding of Cat316, WFA, and Sema3A (**A**) Cat316 specifically recognizes a GalNAc residue at the non-reducing terminal. In contrast, WFA can bind to both terminal and internal GalNAc residues. Our analysis suggests that Sema3A may bind to non-sulfated CS structures located adjacent to the non-reducing terminus. (**B**) On the left side, WFA- and Cat316-positive PNNs are schematically shown. WFA-positive PNNs form a meshwork surrounding the PV cells. Inside the WFA-positive PNNs, Cat316-positive PNNs are distributed in a punctate pattern rather than a meshwork structure. PNN effectors, such as Sema3A and OTX2, are co-localized with Cat316-positive CS chains. On the right side, a biosynthetic model in which Cat316-positive CS chains are synthesized in a ChGn1-dependent manner is shown.

WFA and Cat316 are considered to recognize CS chains attached to aggrecan core proteins [27,30]; however, WFA- and Cat316-positive PNNs are spatially segregated and form microdomains in a single PNN; Cat316-positive CS chains are distributed inside WFA-positive PNNs (Figure 4A and Figure 6B). Moreover, Ueno et al. reported that WFA-positive PNNs and Cat316-positive PNNs do not overlap [32]. Notably, OTX2 and Sema3A are co-localized with Cat316-positive CS chains (Figure 5) [26]. These findings suggest that the differential glycosylation of aggrecans controls the formation of microdomains in a single PNN and the selective binding of OTX2 and Sema3A. How are Cat316-positive CS chains-carrying aggrecans generated? We discuss this phenomenon from biosynthesis and degradation viewpoints. The putative biosynthetic mechanism of Cat316-positive CS chains is shown in Figure 6B. Chondroitin GalNAc transferase-1 (ChGn-1 or CSGalNAcT1) is a key enzyme involved in the initiation of CS biosynthesis [33,34]. Half of the CS chains in the adult cerebrum are synthesized in a ChGn-1-dependent manner [26]. In addition, we previously reported that both the formation of Cat316-positive PNNs and the accumulation of OTX2 are reduced in mice lacking ChGn-1 [26]. Thus, we suppose that ChGn-1 facilitates the synthesis of Cat316-positive CS chains that are involved in the control of OTX2. A recent study has shown that CS synthesized by ChGn-1 is needed for the onset and offset of the critical period-plasticity in visual cortices [14]. In cartilages, specific short-chain CS species are produced in a ChGn-1-dependent manner [35], implying that Cat316 recognizes specific short-chain CS species. These findings raise the possibility that aggrecan glycoforms contribute to the formation of microdomain structures in a single PNN; that is, Cat316-positive CS chains-carrying aggrecans are involved in the formation of the “not condensed” inner PNNs rather than of the “classic lattice-like” outer PNNs formed by WFA-positive CS chains-carrying aggrecans [26]. This difference probably causes a distinct binding property to Sema3A and OTX2. In addition, it is likely that OTX2 and Sema3A binding sites located at the non-reducing terminus of CS chains become accessible in Cat316-positive PNNs, whereas they may be inaccessible as they are buried in the WFA-positive PNN meshwork. Next, we discuss the hypothesis that PNN remodeling by aggrecanases and matrix metalloproteinases (MMPs) controls the binding of Cat316-reactive CS chains to Sema3A. Aggrecanases, the collective term for aggrecan-degrading enzymes, are metalloproteinases that belong to a disintegrin and metalloproteinase with thrombospondin motifs (ADAMTS), a family of extracellular proteases. MMPs and ADAMTS, which are families of metalloproteinases, are considered the most important mediators of aggrecan degradation [36,37,38]. Recent studies have identified MMP-16 as a schizophrenia risk gene [39], and MMP-9 and ADAMTS-5 are implicated in schizophrenia and cerebral cavernous malformations, respectively [40,41]. Remarkably, all of these proteases are involved in the degradation of aggrecans. Thus, we speculate that aggrecans carrying Cat316-positive CS chains are derived from the degradation products of aggrecans. Aggrecans in PNNs are degraded during rapid turnover and their degradation products may be released inside WFA-positive PNNs; does aggrecan glycosylation affect their susceptibility to MMPs and aggrecanases? We have reported the possibility that overexpression of 6-sulfation renders aggrecan more susceptible for degradation by ADAMTS-5, which is expressed in the adult mouse brain [42]. In addition, previous reports have shown that aggrecan glycosylation has a role as a regulator of aggrecanase-mediated cleavage at the Glu^373^-Ala^371^ bond [43]. Interestingly, chondroitinase ABC-treated aggrecans are reportedly cleaved at several additional aggrecanase-susceptible sites, except for the Glu^373^-Ala^371^ bond [43]; in addition, keratan sulfate (KS) was suggested to be important for the recognition of this cleavage site by aggrecanase [43]. Our preliminary results indicate that the modification of brain PGs with KS was increased in ChGn-1 knockout mice compared with that in wild type mice. Thus, it appears that ChGn-1 controls the degradation of aggrecans through Cat316-positive CS chain and KS chain modifications. However, there is no direct experimental evidence for the involvement of ChGn-1 and aggrecanases in the generation of Cat316-positive CS chains and in the PNN remodeling. Further studies are needed to elucidate the aggrecan degradation mechanism via ChGn-1-dependent glycosylation. What is the biological significance of the binding of Sema3A and OTX2 to aggrecan degradation fragments? For example, it might be needed for the internalization of OTX2 by PV cells. Beurdeley et al. reported that CS chains in PNNs facilitate the persistent internalization of Otx2 by PV cells to maintain the closure of critical periods, although the specific target PGs of OTX2 have yet to be identified [19]. In cartilages, aggrecan G1 domain fragments are internalized through a CD44-hyaluronan interaction. In addition, OA cartilage contains several GAG-containing aggrecan degradation fragments with the G1 domain; however, the cellular uptake of these fragments remains unclear [44]. Thus, OTX2 may be incorporated in PV neurons together with GAG-containing aggrecan degradation fragments with the G1 domain. In addition, cleavage by aggrecanase may allow the CS-rich C-terminal fragments of aggrecans to move freely as aggrecans are anchored by binding to hyaluronic acids through the N-terminal G1 domains. Consequently, aggrecan degradation fragments are able to bind to CS receptors on the cell surface. So far, several cell surface proteins reportedly are putative receptors for GAG chains. For example, annexins interact with GAGs [45,46,47]. In particular, annexin A2 is a multifunctional protein involved in endocytosis, exocytosis, membrane domain organization, mRNA transport, and DNA replication [48], and is expressed in GABAergic interneurons that contain PV neurons [49]. It is possible that OTX2 is internalized in PV neurons via annexin A2 in a CS-dependent manner. It would be fascinating to elucidate whether aggrecan degradation is involved in the internalization of OTX2 by PV cells in the future.

What would be the functional implication of the presence of Sema3A in Cat316-positive PNNs? Although the role of Sema3A in PNNs remains unclear, Vo et al. have indicated that Sema3A may restrict axon growth and plasticity through interactions with CS-PGs [31]. They observed that the Sema3A receptor components Plexin A1 and A4 are concentrated on the plasma membrane of interneurons. In addition, Yang et al. demonstrated that blocking the interactions between Cat316-positive CS chains and Sema3A restores memory in tauopathy-induced neurodegeneration [21]. These findings suggest that the preferential binding of Sema3A to Cat316-positive PNNs regulates Sema3A-Plexin-mediated signaling between PNNs and the soma of inhibitory neurons; however, whether there are differences between Cat316-positive PNNs and WFA-positive PNNs in controlling Sema3A-Plexin-mediated signaling remains unclear.

## 5. Conclusions

Previous study has shown that Sema3A interacts preferentially with CS-E in a concentration-dependent manner. In this study, it is shown that Sema3A proteins still accumulated in PNNs even in the absence of CS-E. Thus, Sema3A proteins may not bind to E units within a CS chain in vivo. In addition, both WFA-positive and Cat316-positive PNNs were not affected by loss of CS-E.

Sema3A-binding CS structures were identified taking advantage of recognition properties of Cat316, which is considered to recognize OTX2- and Sema3A-binding sites. As a result, it is suggested that Sema3A preferentially bound to non-sulfated GalNAc residues located adjacent to the non-reducing terminus.

## Figures and Tables

**Table 1:** Primers used in this study.

Assay ID	Reporter Dye	Context Sequence	Gene Symbol	Gene Name	Species	NCBI Gene Reference
Mm00545794_m1	FAM	5′-CTCAGAAGAAGTTCCAGACCATGAC-3′	Acan	aggrecan	Mus musculus	NM_007424.2 L07049.1
Mm00476090_m1	FAM	5′-TCTACTGCTTCCGAGACTCTGCCCA-3′	Bcan	brevican	Mus musculus	NM_007529.2 NM_001109758.1 X87096.1 BC052032.1 AK144405.1
Mm00484007_m1	FAM	5′-AGGAAGATCCCACAGATCCCTGCGA-3′	Ncan	neurocan	Mus musculus	NM_007789.3 X84727.1 BC065118.1
Mm01283063_m1	FAM	5′-CACTGGCTGTGGATGGTGTTGTGTT-3′	Vcan	versican	Mus musculus	NM_172955.1 NM_001134474.1 NM_001134475.1 NM_019389.2 NM_001081249.1 D28599.1 D32040.1 D16263.1 BC021652.1 AK014525.1 AK034871.1 BC096495.1 AK135324.1 AK163927.1 AK165069.1
Mm00478484_m1	FAM	5′-GTCCTACACAGGAGCACTAAATCAA-3′	Ptprz1 (Pcan)	protein tyrosine phosphatase, receptor type Z, polypeptide 1 (Phosphacan)	Mus musculus	NM_001081306.1 AJ133130.1 AK081698.1 BC157965.1 BC151071.1
Mm01179833_m1	FAM	5′-AAGGAGGTGCTCGTAGCCGTGATCG-3′	Sdc3	syndecan 3	Mus musculus	NM_011520.3 BC054795.1 AK129153.1 BC062093.1 AK150120.1 AK155127.1
Mm99999915_g1	FAM	5′-GGTGTGAACGGATTTGGCCGTATTG-3′	Gapdh	glyceraldehyde-3-phosphate dehydrogenase	Mus musculus	NM_008084.2 M32599.1 BC082592.1 BC083079.1 BC083080.1 BC083065.1 BC083149.1 BC085315.1 BC085274.1 BC085275.1 AK191938.1 AK192040.1 AK201736.1 BC091768.1 BC092252.1 BC092264.1 BC092294.1 BC093508.1 BU5

## Supplementary Materials

The following are available online at https://www.mdpi.com/2218-273X/10/11/1499/s1, Figure S1: The expression level of chondroitin sulfate proteoglycans was hardly affected by the loss of E units.
