# Supplementary material for: Reconsideration of the Semaphorin-3A Binding Motif Found in Chondroitin Sulfate Using Galnac4s-6st-Knockout Mice
Source: Biomolecules. 2020 Oct 30;10(11):1499. doi: 10.3390/biom10111499 (PMC7694144; doi:10.3390/biom10111499)

Supplementary Figure S1

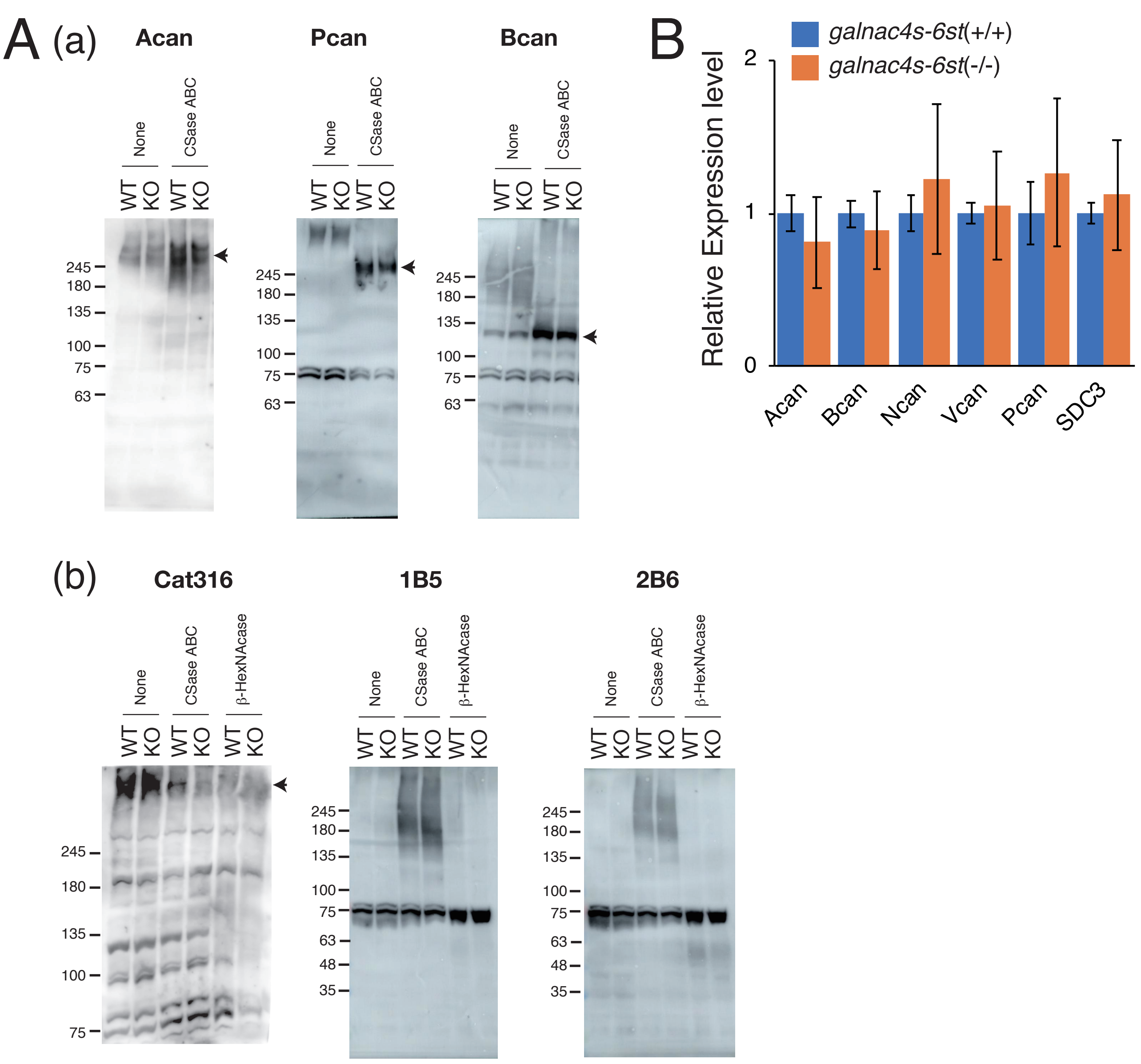

Supplementary Figure S1. The expression level of chondroitin sulfate proteoglycans was hardly affected by the loss of E units

A, (a) Brain extracts prepared from galnac4s-6st(+/+) (WT) and galnac4s-6st(-/-) (KO) mice treated with or without chondroitinase ABC were analyzed by immunoblotting using antibodies against aggrecan, phosphacan, and brevican. Proteoglycan core proteins revealed after digestion with chondroitinase ABC are indicated by arrowheads. (b) Chondroitin sulfate proteoglycans were detected using anti-CS antibodies, Cat316, 1B5, and 2B6. The white triangle indicates proteoglycans carrying Cat316-reactive CS chains. B, The gene expression levels of six types of proteoglycans were examined by real-time PCR.

Spplementary Materials

Original Images for Blots shown in Supplementary Figure S1

Acan

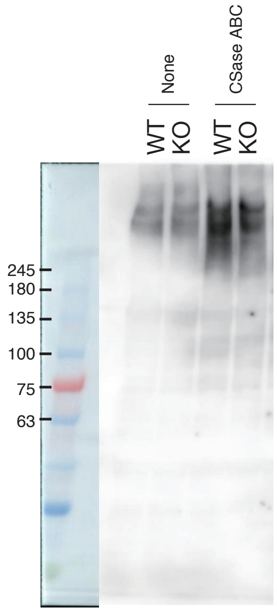

Pcan

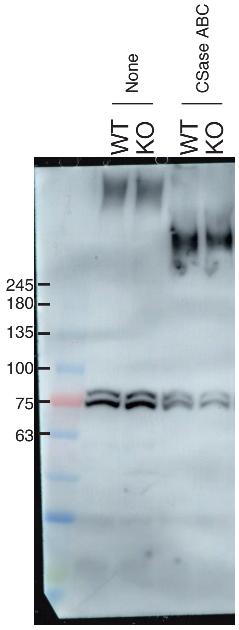

Bcan

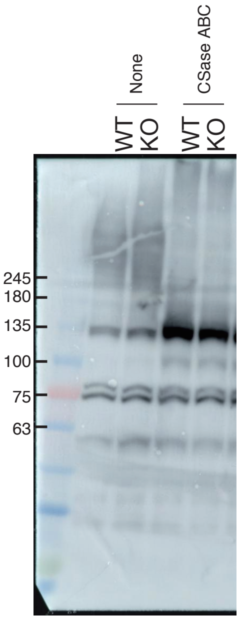

Cat316

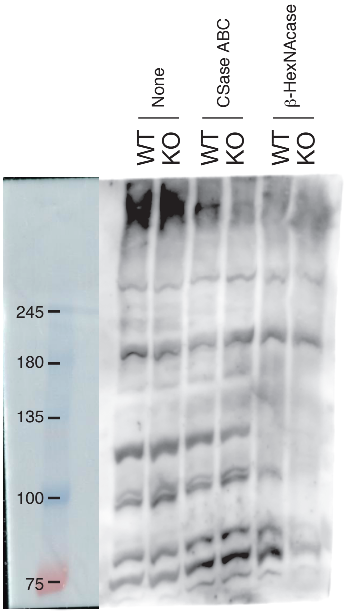

1B5

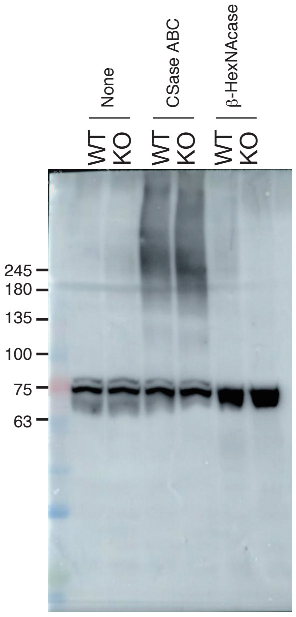

2B6

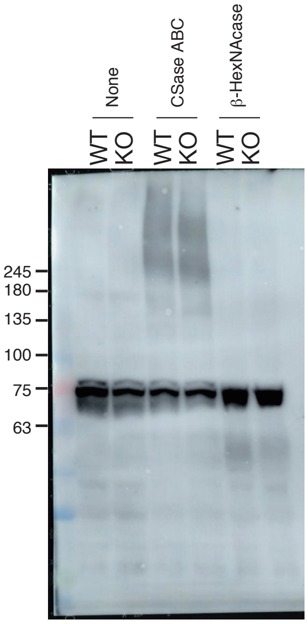

Supplement: Supplementary file 1 [file biomolecules-10-01499-s001.pdf]
